# Comparison of anxiety and child-care education characteristics of mothers who have children with or without speech delays^☆^

**DOI:** 10.1016/j.bjorl.2017.12.004

**Published:** 2018-01-05

**Authors:** Talih Özdaş, Ayşe Sanem Şahlı, Behiye Sarıkaya Özdemir, Erol Belgin

**Affiliations:** a Adana Numune Education and Research Hospital, Otolaryngology Clinic, Adana, Turkey; b Hacettepe University, Vocational School of Health Services, Hearing and Speech Training Center, Ankara, Turkey; c Ankara Child Health and Disease Hematology, Oncology Educational Research Hospital, Department of Pediatric, Ankara, Turkey; d Istanbul Medipol University, Faculty of Health Sciences, Audiology Department, Istanbul, Turkey

**Keywords:** Mother, Anxiety level, Speech delay, Beck anxiety scale, PARI, Mãe, Nível de ansiedade, Atraso na fala, Escala de ansiedade de Beck, PARI

## Abstract

**Introduction:**

Speech delay in a child could be the cause and/or result of the emotional disorder. The child rearing attitude that the parents have accepted could have both positive and negative effects on the personality of the child.

**Objective:**

The current study aimed to investigate the sociodemographic features and the mothers’ anxiety of children with speech delay.

**Methods:**

One hundred five mothers with children aged between 3 and 6 years with speech delays were included in the patient group, and 105 mothers who have children aged between 3 and 6 years with normal speech and language development were included in the control group. An information form questionnaire including demographic characteristics, the Family Life and Childrearing Attitude Scale (PARI – Parental Attitude Research Instrument) and Beck anxiety scale were requested from all mothers in the patient and the control groups.

**Results:**

In the current study, there was a significant difference between the groups in terms of gender (*p* = 0.001). According to Parental Attitude Research Instrument, the mean of mothers of the children with speech delays was higher than the mean of mothers of normal children in terms of the answers to overprotective mother aspect (*p* < 0.01). The mothers of children with speech delays had more overprotective motherhood attitudes; however, the difference in terms of the answers to the aspects of democratic attitude and provision of equality, refusal to be a housewife, husband-wife conflict, and suppression and discipline were not statistically significant. The Beck anxiety scale, a significant difference was detected between the two groups (*p* < 0.01). It was found that the mothers of children with speech delays had more severe levels of anxiety.

**Conclusion:**

The social structure of the family, the attitudes and the behaviors of the mother, and the anxiety levels of the mothers have important effects on child development. Thus, it is necessary to perform further studies related to speech delays, in which many factors play a role in the etiology.

## Resumo

**Introdução:**

O atraso da fala em uma criança pode ser a causa e/ou o resultado de distúrbio emocional. As atitudes tomadas pelos pais na educação da criança podem ter efeitos positivos e negativos sobre a personalidade infantil.

**Objetivo:**

Investigar as características sócio-demográficas e a ansiedade das mães de crianças com atraso na fala.

**Método:**

Foram incluídas no grupo de pacientes 105 mães com crianças entre 3 e 6 anos de idade e atrasos na fala, e no grupo controle 105 mães com crianças na mesma faixa etária e desenvolvimento normal da fala e da linguagem. Foi solicitado às mães de ambos os grupos o preenchimento de informações em um formulário, incluindo características demográficas, o instrumento *Family Life and Child Rearing Attitude Scale* (PARI – *Parental Attitude Research Instrument*) e o inventário de ansiedade Beck.

**Resultados:**

No estudo atual, houve diferença significativa entre os grupos em relação ao gênero (*p* = 0,001). De acordo com o instrumento *Parental Attitude Research Instrument*, a média das mães das crianças com atrasos na fala foi maior do que a média das mães de crianças normais em relação às respostas sobre superproteção materna (*p* < 0,01). As mães de crianças com atrasos na fala apresentaram mais atitudes de proteção materna excessiva; no entanto, a diferença das respostas sobre atitudes democráticas e igualitarismo, recusa do papel de dona de casa, conflito marital e disciplina rígida não foram estatisticamente significantes. No inventário de ansiedade de Beck foi encontrada uma diferença significativa entre os dois grupos (*p* < 0,01). Verificou-se que as mães de crianças com atrasos na fala apresentavam níveis mais graves de ansiedade.

**Conclusão:**

A estrutura social da família, as atitudes, o comportamento e os níveis de ansiedade das mães têm efeitos importantes no desenvolvimento da criança. Conclui-se, portanto, a necessidade de realizar estudos adicionais relacionados aos atrasos na fala, nos quais diversos fatores desempenham um papel etiológico.

## Introduction

Speech delay is defined as inability to demonstrate speech-language skills that is expected according to the age. The prevalence of speech delay in children between aged 2 and 7 varies at the range of 2.3–19%. Speech delay throughout school period and continuing even at adulthood is observed in 5–8% of the children who had speech delay during preschool period.^1^

There are many factors that could cause speech delays. The most frequently reported risk factors for speech delays are positive family history, male gender, a history of prematurity, and low birth weight. Other risk factors that are thought to be less associated are low education level of the parents, history of childhood disease, late birth sequence, older parents, low socioeconomic status, and the presence of a large family.^2^, ^3^

Anxiety disorders and depression are the most common psychological disorders in the general population. The prevalence of anxiety disorders in the urban population was 9.1% in males and as 18.1% in females. Speech delay in children affects the emotional status of the mother, and the emotional disorder or depression of mother could also negatively affect speech development in children. Speech delay in a child could be the cause and/or result of the emotional disorder.^4^ The child rearing attitude that the parents have accepted could have both positive and negative effects on the personality of the child.

The current study aimed to investigate the etiological factors of speech delay in children in terms of sociodemographic features and to compare the mothers of children with speech delay and the mothers of normal children in terms of childrearing attitudes and anxiety levels.

## Materials and methods

### Subject recruitment

The participants were informed about the study, and their verbal and written informed consents were obtained. Local ethics committee approved the study protocol, and study was conducted in accordance with the human rights and experimental ethics (Project Number: KA15/168).

A total of 105 children who were admitted to the audiology sound and speech disorders unit and ear-throat-nose policlinic with complaints of speech delay, and who were diagnosed with speech delays as a result of speech and language assessment (the results of evaluation as normal receptive language and delayed expressive language) and their mothers; and 105 children with normal speech and their mothers were included in the study.

Psychosocial factors are thought to be the leading cause of etiological factors in children with speech delay who are included in the study. These factors include lack of speech stimuli, inadequate social communication in the family, and long-term use of television and computers. the control group was randomly selected from children aged 3–6 years who were attending a preschool institution in a region with the same socioeconomic characteristics. The researchers interviewed their parents and their teachers and learned about their language and speech development. Children suspected of having language and/or speech delay are not included in the study.

For both groups, our inclusion criteria is children aged 3–6 years who were not at high risk of developmental delay or suspected of having developmental delay and our exclusion criterias are children born preterm (gestational age <37), with low birth weight (<2500 g) and children with other know disorders that may be associated with or affect development. After the parents of the participant children have been informed about the process, participant permission to the study has received by signing voluntary consent form.

### Physical examinations

Physical examinations of the ear–throat–nose system and hearing assessments were performed in all children, and the children who had hearing levels between (−10) and (+15) dB (decibel) were accepted as normal and included in the study. The children with mental retardation, hearing loss, history of trauma, complications related to previous childhood disease, and neurological disease were not included to the study.

### The speech and language assessment

The speech and language assessment were performed with the Preschool Language Scale-Fifth Edition (PLS-5). PLS-5, which is widely used around the world, is a language assessment test that has been prepared and implemented by Zimmerman, Steiner, and Pond in 2011. The test was brought to Turkey in 2013 and the Turkish translation, its adaptation for Turkish children and its validity and reliability is done with 1320 children between 0–7 years and 11 months by Sahli, A.S and Belgin, E. It is used to determine whether there is delay in language development, to evaluate receptive and expressive language development.^5^

### The demographic information

Demographic information form consists of questions that interrogate not only demographic characteristics of the mother (age, occupation, educational status, number of children, economic status, etc.), but also information related to the child (age, which child, etc.), and various developmental statuses including language and speech.

### Parental Attitude Research Instrument, (PARI) [Family Life and Childrearing Attitude Scale (FLCAS)]

PARI, developed by Schaefer and Bell in the United States in 1958,^6^ was adapted in 1978 by Guney le Complete, Ayhan le Complete, and Serap Ozer.^7^ The scale consists of 60 items and five subitems, including excessive motherhood/overprotection, refusal of the role of being a housewife, offering democratic approach/equality, marriage conflict/disagreement, and strict discipline.^6^, ^7^ Reliability coefficients were reported to be 58 and 88, and the alpha reliability coefficient was 64. The second adaptation has been performed by Kucuk (1990).^8^

### Beck anxiety scale (BAS)

The BAS was developed by Beck et al. in 1988, due to the need for a scale that differentiates anxiety and depression.^9^ It measures the severity of anxiety symptoms that the individual experiences. The BAS is a scale that contains 21 items, four of which are self-assessment items and graded according to a Likert scale (0–3) and is completed by the patient. The score interval is 0–63. The high total score in the scale demonstrates the severity of the anxiety that the individual experiences. A score of 0–8 indicates the absence of anxiety, a score of 8–15 indicates a mild degree of anxiety, a score of 16–25 indicates a moderate degree of anxiety, and a score of 26–63 indicates severe anxiety. The validity-reliability studies were performed by Ulusoy, Sahin, and Erkmen.^10^

### Data analysis

The SPSS 17.0 program was used for statistical analysis. Descriptive statistics were used for the mean, standard deviation, median, and minimum and maximum values. The Chi-square test was used to analyze the classified data. The Student's *t*-test was used to compare the mean of the two groups, and the one-way ANOVA test was used to compare the mean of more than two groups. *p* < 0.05 was accepted as statistically significant.

## Results

### Recruitment

In the current study, the mothers of 105 children with speech delays and the mothers of 105 normal children were compared in terms of childrearing attitudes and anxiety levels of mothers, and the causes of speech delay were examined in terms of sociodemographic characteristics; a controlled comparison was performed. The demographic characteristics of the mothers and children in both groups are summarized in Table 1.

**Table 1:** Demographic characteristics of mothers in the study and control groups.

	Group I		Group II		*p*^a^
	*N*	%	*N*	%	
*Gender of children*					
Boy	75	71.4	50	47.6	**0.001**
Girl	30	28.6	55	52.4	

*Maternal education level*					
Less than high school graduate	43	41.0	56	53.3	**0.008**
High school graduate	32	30.5	37	35.2	
College graduate	30	28.6	12	11.4	

*Mother's work status*					
Housewife	83	79	88	83.8	0.478
Working	22	21	17	16.2	

*Parent's income level*					
€/month <500	78	38.4	40	38.1	**0.000**
€/month 500–1000	–	–	10	9.5	
€/month 1000–2000	83	40.9	43	41.0	
€/month >2000	42	20.7	12	11.4	

*Family structures*					
Nuclear family	105	100	68	65	**0.000**
Extended family	–	–	32	31	
Step family	–	–	5	4	

*Birth order*					
First child	22	21	38	36.5	**0.000**
Second child	83	79	50	48.1	
Third child	–	–	6	5.8	
And after the fourth child	–	–	10	9.6	

*N*, number of individuals; %, percentage; Group I, mothers of children with speech delays (*n* = 105); Group II, mothers of children with normal speech (*n* = 105).

a *p*-Value = 0.05, considered significant. Bolds are statistically significant.

A total of 105 mothers of children with speech delays, including 75 males (71.4%) and 30 females (28.6%), constituted the patient group. A total of 105 mothers of normal children, including 50 males (47.6%) and 55 females (52.4%), constituted the control group. The difference between the groups in terms of gender was statistically significant using the Chi-square test (*p* = 0.001) (*p* < 0.01).

The age of the children in the patient group ranged between 36 and 72 months and the mean age was 55.18 ± 17.29 months. The age of children in the control group ranged between 36 and 63 months and the mean age was 45.42 ± 8.51 months.

### The responses to the BAS between study and control groups

According to the responses to the BAS, anxiety was not detected in 46 mothers (43.8%), mild anxiety was detected in 45 mothers (42.9%), moderate anxiety was detected in 7 mothers (6.7%), and severe anxiety was detected in 7 mothers (6.7%) among the 105 mothers of children with speech delays in the patient group.

According to the responses to the BAS, anxiety was not detected in 57 mothers (54.3%), mild anxiety was detected in 32 mothers (30.5%), and moderate anxiety was detected in 16 mothers (15.2%) in the 105 mothers of children with normal speech development.

When the mothers of children with speech delays and those with normal speech development were statistically compared according to the responses to the BAS, a statistically significant difference was detected between the two groups in the Chi-square test (*p* < 0.01) (Table 2).

**Table 2:** Distribution of anxiety levels of mothers in the control and patient groups according to scores in the BAS.

	Group I		Group II	
	*N*	%	*N*	%
Insignificant	46	43.8	57	54.3
Mild	45	42.9	32	30.5
Moderate	7	6.7	16	15.2
Severe	7	6.7	–	–

*N*, number of individuals; %, percentage; Group I, mothers of children with speech delays (*n* = 105); Group II, mothers of children with normal speech (*n* = 105).

### The responses to the PARI between study and control groups

When the statistical results of the responses to the subitems of PARI scale was compared in mothers of children with speech delays and with normal speech development (Table 3).

**Table 3:** Distribution of mothers according to scores in the subdimensions of PARI in the patient and the control groups.

	Group I	Group II	*p*^a^
Overprotection	42.4 ± 4.69	40.8 ± 3.82	**0.008**
Democratic/Equality	24.0 ± 5.26	22.9 ± 5.86	0.179
Strict Discipline	43.30 ± 4.99	42.05 ± 4.85	0.068
Husband-wife Conflict	16.19 ± 3.45	15.50 ± 3.90	0.179
Refusal to be a Housewife	30.88 ± 9.35	29.94 ± 9.39	0.467

SD, standard deviation; Group I, mothers of children with speech delays (*n* = 105); Group II, mothers of children with normal speech development (*n* = 105). Results were given as mean ± SD.

a *p*-Value 0.05 considered significant. Bolds are statistically significant.

The mean score of mothers in the patient group according to the answers to Overprotective Motherhood was 42.4 ± 4.69. The mean score in the control group was 40.8 ± 3.82. There was a statistically significant difference between the two groups according to the Student's *t*-test (*p* < 0.01). The mean score of the mothers of children with speech delays was higher than the mean score of the mothers of children with normal speech. The mothers of children with speech delays had a more protective motherhood attitude.

The mean score of the mothers according to the answers to Offering Democratic Attitudes and Equality dimension was 24.0 ± 5.26 in the patient group and it was 22.9 ± 5.86 in the control group. There was no statistically significant difference between the two groups in terms of mean score, according to the Student's *t*-test (*p* > 0.05).

The mean score of the mothers according to the responses to the Refusal to be a Housewife dimension was 30.88 ± 9.35 in the patient group and it was 29.94 ± 9.39 in the control group. There was no statistically significant difference between the two groups according to the Student's *t*-test (*p* > 0.05).

The mean score of the mothers according to the responses to the Husband-wife Conflict dimension was 16.19 ± 3.45 in the patient group and it was 15.50 ± 3.90 in the control group. The difference between two groups was not statistically significant according to the Student's *t*-test (*p* > 0.05).

The mean score of the mothers according to the responses to the Suppression and Discipline was 43.30 ± 4.99 in the patient group and 42.05 ± 4.85 in the control group. Similarly, the difference between two groups was not statistically significant according to the Student's *t*-test (*p* > 0.05).

## Discussion

Although the etiology of speech delay is currently unknown, many variables have been described as potential risk factors. The most important risk factors for speech delay are male sex, factors associated with socioeconomic disadvantage, family history of developmental speech-language disorder, and persistent otitis media.^11^ U.S. Preventive Services Task Force (USPSTF) was unable to develop a list of specific risk factors to guide primary care providers in selective screening. The most consistently reported risk factors included a family history of speech and language delays, male sex, and perinatal factors, such as prematurity and low birth weight.^12^ The study of Horwitz et al.,^13^ which included 1189 children with speech delays, in 2003, reported that 51% of the children were male and 49% was female. The study of Zubrick et al.,^14^ conducted in 2007 and including a total of 1766 children, reported 1528 children with normal speech development and 238 children with speech delays. It reported that 47.6% of the children that had normal speech and 70.8% of the children that had speech delays were male and the difference was statistically significant.

In the current study, among the children with speech delays, 75 (71.4%) were male, 30 (28.6%) were female, whereas among the children with normal speech development, 50 (47.6%) were male and 55 (52.4%) were female. The difference between the groups in terms of gender was statistically significant (*p* = 0.001). In comparison to the literature, speech delays were more common in males compared to females in the current study.

In the study of Sahli,^15^ which was conducted in 20 children with hearing loss and their fathers in 2011, when the mean score of the fathers according to the PARI subdimensions were examined, the scores of the dimension of the overprotective approach were significantly higher in fathers of children with hearing loss (42.76 ± 5.59) compared with the fathers of children without hearing loss (40.16 ± 6.55) (*p* < 0.01). It was emphasized that this result was an indicator of more protective behavior by the fathers of children with hearing loss. Furthermore, the fathers of children with hearing loss had lower scores in the democratic/equality and strict discipline dimensions, and these values were statistically significant (*p* < 0.01). Thus, this was emphasized as an indicator that fathers of children with hearing loss demonstrate less democratic behavior and lesser discipline.

When the mothers of children with speech delays and those with normal speech development were compared according to the responses to the subdimensions of the PARI. The mean score of the mothers of children with speech delays was higher than the mean score of the mothers of children with normal speech development, according to the responses to the Overprotective motherhood dimension; the difference was statistically significant (*p* < 0.01). The mothers of children with speech delays demonstrated more protective motherhood attitudes.

The difference in the mean score according to the responses to the Democratic Attitudes and Equality, Refusing to be a Housewife, Husband-Wife Conflict, and Suppression and Discipline dimensions were not statistically significant in mothers of children with speech delays and those with normal speech development.

It is known that the attitude of the family has both positive and negative effects on all developmental fields of the children. The current study revealed that the mothers of children with speech delays demonstrated more protective attitudes and behaviors. Mothers with this type of attitude could not understand that their children are individuals different from themselves and these children need to gain their independence. These parents always behave as if their children were smaller than they. They feed the child that eventually feeds himself; they clothe the child that eventually clothes by himself. Thus, as all needs of the children are met by their parents, it is thought that these children have no need to speak.

The Depression Anxiety Stress Scale (DASS) was used in the study of Zubrick et al., and normal, mild, and moderate-severe degrees of anxiety were detected in 94.5%, 1.9%, and 3.7% of the mothers of children with normal speech, respectively. The degree of anxiety was normal, mild, and moderate-severe in 93.3%, 2.1%, and 4.6% of the mothers of children with speech delays, respectively. No statistically significant difference was detected between the groups.^14^

The study of Cırpar et al.,^16^ conducted in 2010, included the mothers of 18 children with speech delays and 20 healthy children. The level of anxiety was evaluated with State-Trait Anxiety Inventory (STAI). The mothers in the patient group had STAI-S scores of 44 ± 13.5 and STAI-C scores of 45.2 ± 9.4, and the mothers in the control group had STAI-S scores of 36.9 ± 9.9 and STAI-C scores of 41.0 ± 5.3. A statistically significant difference was detected in the mean STAI-S scores of the mothers of children with speech delays and the mothers of healthy children (*p* = 0.046).

In the current study, among 105 mothers of children with speech delays, anxiety was not detected in 46 mothers (43.8%), mild anxiety was detected in 45 mothers (42.9%), moderate anxiety was detected in 7 mothers (6.7%), and severe anxiety was detected in 7 mothers (6.7%) according to the BAS. Among 105 mothers of children with normal speech, anxiety was detected in 57 mothers (54.3%), mild anxiety was detected in 32 mothers (30.5%), and moderate anxiety was detected in 16 mothers (15.2%). When the answers to the BAS were statistically compared, a significant difference was detected between the mothers of children with speech delays and the mothers of children with normal speech (*p* < 0.01). Furthermore, the current study revealed that the severity of anxiety in mothers of children with speech delays was greater. Speech delays in children negatively affect the level of anxiety in mothers and the high level of anxiety in mothers might also negatively affect speech development in children. It is thought that speech delays in children could be both the cause and/or the result of the anxiety disorder in the mother. The small sample size is the limitation to this study that should be addressed.

## Conclusions

When the mean scores of mothers of children with speech delays and the mothers of healthy children were statistically compared according to the responses to the subdimensions of PARI, the mothers of children with speech delays demonstrated more protective attitudes and behaviors. The mothers who had such attitudes and behaviors met the requirements of their children without the need to speak; therefore, speech might have been delayed in children who had mothers with such behavior.

When the BAS of the mothers of children with speech delays were compared to the mothers of normal children, a significant difference was detected between the two groups, and the mothers of children with speech delays had more severe anxiety. The anxiety might be a result of speech delays in the children and the high level of anxiety in the mothers might be the reason for the speech delay. Further studies are required to investigate speech delays and its relationship to the family and society structure in our country.

## Conflicts of interest

The authors declare no conflicts of interest.
